# O.3.3-6 Brazilian immigrant parents' preferences for content and intervention modalities for the design of a family-based intervention to promote their preschool-age children’s healthful energy balance-related behaviors

**DOI:** 10.1093/eurpub/ckad133.161

**Published:** 2023-09-11

**Authors:** Thais Caires, Qun Le, Mary L Greaney, Ana Cristina Terra de Souza Lindsay

**Affiliations:** University of Massachusetts Boston; University of Massachusetts Lowell; University of Rhode Island; thais.rocha001@umb.edu; University of Massachusetts Boston

## Abstract

**Purpose:**

Childhood obesity is a complex public health problem disproportionally affecting racial/ethnic minority populations and children in immigrant families. Promoting healthful energy balance-related behaviors (EBRBs) in young children, a fundamental obesity preventative strategy, is critical for addressing health disparities in childhood obesity among ethnic minority and immigrant populations. Accumulating evidence indicates that unhealthful EBRBs, including poor eating behaviors (e.g., high consumption of unhealthy foods and sugar-sweetened beverages [SSBs]), inadequate physical activity (PA), excessive sedentary behavior/screen time, and short sleep duration, increase the risk of childhood overweight and obesity. Brazilians are a rapidly growing ethnic immigrant population in the United States (U.S.) and currently, there is a lack of childhood obesity prevention interventions addressing the needs of Brazilian preschool-age children.

**Methods:**

Guided by the family ecological model, this formative cross-sectional study assessed the preferences (content, intervention modality, and language) of 52 individual Brazilian immigrant families (27 mothers, 25 fathers) for the development of a family-based intervention to promote healthful energy balance-related behaviors (EBRB).

**Results:**

Overall, 85% or more of parents reported being interested or very interested in content related to five (increase fruits and vegetables, reduce unhealthy foods and sugar-sweetened beverages, increase physical activity, and reduce screen time) out of the seven assessed EBRBs. Group sessions delivered by community health workers (CHWs), email, and text messages were the preferred intervention modalities with most parents (71.2%) indicating a preference for content delivered in Portuguese. Interventions integrating multiple components such as workshops offered by CHWs, text messaging using SMS and WhatsApp, and printed materials should all be considered.

**Conclusions:**

Future steps should include investigating different communication channels and their integration into a pilot test of a linguistic and cultural-sensitive family-based intervention to promote healthful EBRBs of preschool-age children in Brazilian families in the U.S.

